# Does Periodontal Disease Influence Tuberculosis Outcomes? Insights from a Scoping Review

**DOI:** 10.5334/aogh.5343

**Published:** 2026-09-07

**Authors:** Prerna Hirkane, Ajay Kumar Verma, Umesh Pratap Verma, Arpita Singh

**Affiliations:** 1 Department of Periodontology, Faculty of Dental Sciences, King George’s Medical University, Uttar Pradesh, Lucknow, India; 2 Department of Respiratory Medicine, Dr Ram Manohar Lohiya Institute of Medical Sciences, Gomti Nagar, Lucknow, India; 3 Department of Pharmacology, Dr Ram Manohar Lohiya Institute of Medical Sciences, Gomti Nagar, Lucknow, India

**Keywords:** periodontitis, tuberculosis, respiratory diseases, periodontal medicine, perio-systemic

## Abstract

*Background:* Periodontitis and tuberculosis (TB) are common chronic infections disproportionately affecting disadvantaged groups. They share behavioural, environmental, and immunological risk factors. Emerging evidence suggests a bidirectional relationship, with TB patients often exhibiting poorer periodontal health and periodontal inflammation potentially exacerbating systemic TB.

*Objective:* To comprehensively map and evaluate the existing evidence regarding the association between periodontal disease and TB outcomes, and to explore the potential influence of periodontal status on TB progression, treatment response, and overall disease outcomes through a scoping review.

*Methods:* This scoping review examined human studies assessing periodontal status in TB patients, including observational studies, clinical trials, and reviews. Searches were conducted in PubMed, Scopus, and Google Scholar, complemented by reference screening. Two independent reviewers applied inclusion criteria, extracted data, and narratively synthesised the findings due to methodological diversity.

*Results:* Out of 1,129 records screened, 8 studies met the inclusion criteria. The majority of observational studies demonstrated a significantly higher prevalence and severity of periodontitis in TB patients relative to controls, characterised by increased probing pocket depths and elevated bleeding on probing. Complementary evidence from recent reviews and microbiome analyses highlights shared inflammatory mediators, immune dysregulation, and alterations in oral microbial communities as potential mechanistic pathways underpinning the association between TB and periodontal tissue destruction.

*Conclusion:* Current evidence supports a consistent but preliminary association between TB and deteriorated periodontal health, indicating complex bidirectional interactions rather than confirmed causality. Routine periodontal screening and oral health counselling integrated into TB management, along with heightened clinical vigilance for TB in unusual periodontal cases, may improve care. Further longitudinal and mechanistic studies are needed to clarify these relationships.

## Introduction

The relationship between oral and systemic health has evolved considerably over the past century. Early in the 20th century, the now-discredited ‘focal infection theory’ proposed that oral infections were responsible for a wide range of systemic diseases, leading to aggressive interventions, such as full-mouth extractions, for disease prevention. Although this theory was later abandoned, it stimulated interest in understanding the biological links between oral infections and systemic health. Subsequent research has demonstrated that the oral cavity harbours a complex and dynamic microbiome that plays a crucial role in maintaining both oral and systemic homeostasis. Disruption of this microbial balance (microbial dysbiosis) in periodontal disease promotes persistent local inflammation and facilitates bacteraemia, allowing periodontal pathogens and their virulence factors to disseminate into the systemic circulation. These events trigger immune modulation and sustained systemic inflammatory responses characterised by the release of pro-inflammatory cytokines, including interleukin (IL)-1β, IL-6, tumour necrosis factor-alpha (TNF-α), and C-reactive protein (CRP), which may contribute to tissue injury at distant sites. The paradigm shifted further in 1996 when Offenbacher introduced the concept of periodontal medicine, emphasising the potential systemic consequences of periodontal inflammation and microbial burden [1]. Since then, accumulating evidence has strengthened the concept of the oral-systemic connection and highlighted the role of periodontal disease beyond the oral cavity. Increasing evidence demonstrates that periodontal inflammation contributes to the pathogenesis of several systemic diseases, including diabetes mellitus, cardiovascular disease, respiratory diseases, rheumatoid arthritis, adverse pregnancy outcomes, and chronic kidney disease.

Periodontal diseases comprise a spectrum of inflammatory conditions affecting the supporting tissues of the teeth. Periodontitis is the most prevalent destructive form characterised by progressive loss of periodontal attachment and alveolar bone. Epidemiological data from 2011 to 2020 indicate that periodontitis remains one of the most prevalent chronic diseases worldwide, affecting approximately 62% of dentate adults, with severe forms present in nearly one-quarter of the individuals [2]. Mechanistically, periodontitis may influence systemic health through pathways such as microbial dissemination, immune dysregulation, chronic inflammatory mediator release (including TNF-α and IL-6), microbial dysbiosis, and shared behavioural, environmental, or genetic susceptibility factors [3].

Tuberculosis (TB), caused by *Mycobacterium tuberculosis*, remains a major global public health challenge despite being preventable and curable. TB exists as **latent tuberculosis infection (LTBI)** or **active disease**, with the latter being transmissible and most commonly affecting the lungs (**pulmonary TB**). However, **extrapulmonary TB** can involve multiple organs, including the oral cavity. Although uncommon, oral TB may present as chronic non-healing ulcers, gingival enlargement, osteomyelitis, or periodontal destruction, often resembling periodontal disease and other oral lesions. These overlapping clinical features make careful differential diagnosis essential, particularly in patients with persistent or atypical periodontal manifestations. According to the World Health Organization’s Global Tuberculosis Report 2024, an estimated 10.7 million individuals developed TB worldwide, highlighting its continued global burden [4, 5]. TB persists as a global priority, and the World Health Organization aims for a 90% reduction in incidence between 2015 and 2035 under the End TB Strategy, highlighting the urgent need for continued research and strengthened public health interventions [6].

Growing evidence suggests possible biological and epidemiological intersections between TB and periodontal disease. Both conditions involve chronic infection, prolonged immune activation, and systemic inflammatory involvement. Moreover, *M. tuberculosis* has demonstrated potential oral manifestations, and immunological mechanisms implicated in TB may exacerbate periodontal breakdown [7]. Periodontal disease and TB share several risk factors, including smoking, diabetes, HIV infection, malnutrition, and socioeconomic disadvantage. Emerging evidence also suggests that periodontitis is associated with respiratory diseases through chronic inflammation, microbial dysbiosis, and immune modulation. However, the relationship between periodontal disease and TB remains unclear. Therefore, this scoping review aimed to map the current evidence, identify knowledge gaps, and highlight priorities for future research [8].

## Methodology

This scoping review was conducted to map and synthesise the existing literature regarding the relationship between periodontitis and TB. By incorporating a diverse array of article types, this review aims to provide a comprehensive overview of the current evidence and identify research gaps within this interdisciplinary field (see Table 1).

**Table 1:** Inclusion and exclusion criteria.

INCLUSION CRITERIA	EXCLUSION CRITERIA
Human studies investigating periodontal status among individuals diagnosed with tuberculosis	Animal and in vitro experimental studies
Epidemiological investigations, including clinical trials, case-control, cross-sectional and cohort studies	Case reports lacking detailed periodontal evaluation
Systematic reviews and meta-analyses primarily focused on immunological pathways or clinical correlations unrelated to direct periodontitis–tuberculosis associations	Studies addressing confounding conditions not directly linked to tuberculosis or periodontitis
Studies published in English language	Non-English language publications

### Search strategy

Comprehensive searches were performed in PubMed, Scopus, and Google Scholar databases using a combination of keywords; periodontitis, TB, perio-systemic, periodontal medicine, periodontitis and respiratory diseases, periodontitis and tb, and oral health and TB from January 2000 to December 2025. Additionally, reference lists of identified articles were hand-searched to capture any pertinent studies not retrieved through database queries. This scoping review was conducted according to the PRISMA-ScR guideline following the Arksey and O’Malley framework, further refined by Levac et al.

### Data selection

The selection of studies was performed independently by two reviewers (PH and AKV) through an initial screening of titles and abstracts to identify potentially eligible articles. Articles deemed relevant were then subjected to full-text evaluation to confirm eligibility according to the predefined inclusion and exclusion criteria. Any discrepancies between reviewers were resolved through consensus discussions, with the involvement of a third reviewer when necessary (PH, AKV, UPV).

### Data extraction

**Figure 1:**
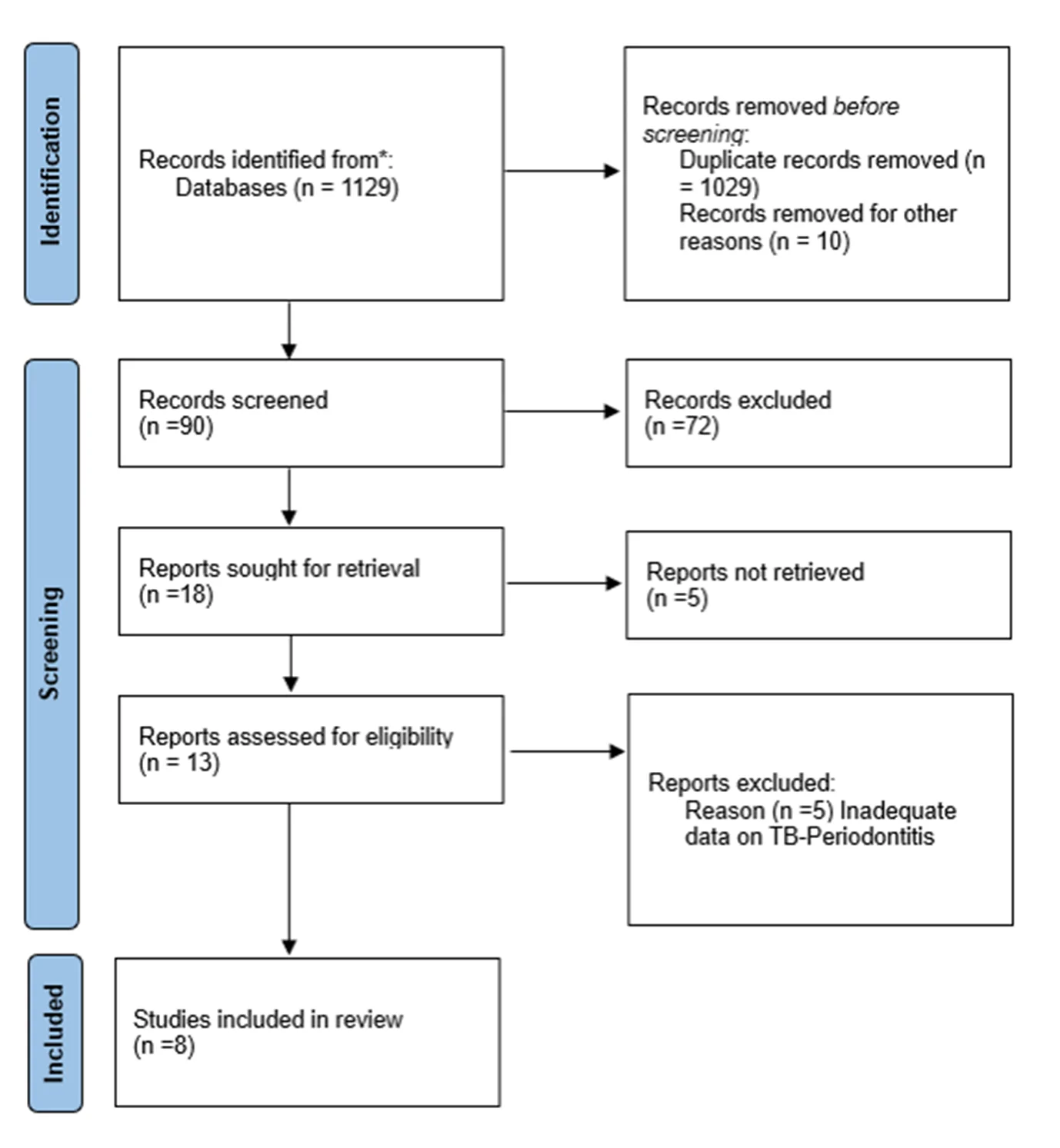
Flowchart illustrating the identification, screening, and inclusion of studies.

Data extraction was independently completed by two reviewers (PH and AS). Extracted data included study design, publication year, sample characteristics, and principal findings relevant to the association between periodontitis and TB (see Figure 1).

### Data synthesis

Extracted data were organised into a unified database for synthesis. Given the expected heterogeneity in study methodologies, populations, and outcome measures, a qualitative narrative synthesis was employed to summarise key results.

## Result

Eight studies met the inclusion criteria, comprising five observational studies (three cross-sectional and two case-control) and three review articles. The characteristics and principal findings of the included studies are summarised in Table 2.

**Table 2:** Study characteristics.

YEAR	AUTHOR	TYPE	FINDINGS	CONCLUSION
2016	Sharma et al. [9]	Case-control	Significant positive correlation was found in PPD 3.08 ± 0.466 and 2.56 ± 0.493 in group I (TB) and group II (non-TB), respectively, and BOP 0.763 ± 0.149 and 0.642 ± 0.222 in group I and group II, respectively, with *P* value ≤0.005*.*	Periodontal status might be linked with TB.
2023	Shahzad et al. [10]	Case-control	Evaluation of periodontal parameters (PPD, gingival bleeding, and GI) showed that **56% of tuberculosis patients had a 6.23-fold higher risk of periodontitis** (95% CI: 4.2–9.1; ***P* < 0.001**). The mean value for PPD and Bitewing X-ray finding was higher in cases (patients suffering from TB) group with a mean score of 3.2 ± 2.07, 1.0 ± 0.1 respectively, as compared to the control group with a mean score of 2.8 ± 1.03 and 1.0 ± 0.0, respectively. The difference between the groups for these parameters showed statistical significance with *P <* 0.001.	Periodontitis and TB have a significant association.
2019	Kumar et al. [11]	Cross-sectional	Among 127 patients suffering from tuberculosis, majority reported with Generalised Periodontitis. The mean loss of attachment was 2.81 ± 1.36 within the range of 0.85–8.7. The mean pocket probing depth was 1.77 ± 0.56 with a range of 0.67–3.53.	Incidence of periodontal disease and oral lesions is higher in tuberculosis patients.
2012	Palakuru et al. [12]	Case-control	The mean difference in attachment loss scores as measured by CPI was 0.48 (*P* = 0.09) and was statistically not significant.	Tuberculosis status did not significantly affect periodontal health; however, the detection of *Mycobacterium tuberculosis* in plaque and saliva may pose a potential clinical risk.
2019	Rastogi et al. [13]	Cross-sectional	TB patients aged 51–60 showed the highest periodontitis prevalence (42%), with males (43%) more affected than females (*P <* 0.01)*.* Based on PIRI (Periodontal index for risk of infectiousness) scores as shown in majority of the population was recorded under high-risk category with highest risk observed in TB patients.	A significant proportion of the study population exhibited periodontitis, with PIRI assessments indicating a predominance of individuals in the high-risk category.
2024	MohanaSundaram et al. [14]	Review	Case reports suggests a potential role of *Mycobacterium tuberculosis* in periodontitis, highlighting a concern amid the rising incidence of tuberculosis, particularly in individuals with HIV and immunocompromised patients.	Tubercle bacilli as a causative of periodontitis are an upcoming concern in oral health.
2025	Noor et al. [15]	Review	Periodontitis may exacerbate systemic conditions like tuberculosis by increasing levels of circulating inflammatory markers, such as C-reactive protein, IL-6, and TNF-α.	The potential association between TB and periodontitis holds significant public health implications.
2025	Ojha et al. [16]	Cross-sectional study	More than two-thirds of the participants had fair plaque control (75.6%), moderate gingivitis (92.2%), and moderate form of periodontitis (73.3%).	Two-thirds of the participants diagnosed primarily with pulmonary disease had periodontitis.

Overall, most observational studies reported a higher prevalence and greater severity of periodontitis among patients with TB compared with healthy controls. Statistically significant increases in probing pocket depth, bleeding on probing, gingival index, and the prevalence of generalised periodontitis were reported in several studies. However, one case-control study found no significant differences in periodontal clinical parameters between TB and non-TB groups.

The review articles consistently described potential associations between TB and periodontitis through shared inflammatory pathways, immune dysregulation, and oral microbiome alterations. Across the included studies, smoking, HIV co-infection, diabetes, and socioeconomic status were commonly identified as potential confounding factors. Detailed study characteristics, outcomes, and conclusions are presented in Table 2.

## Discussion

The potential association between periodontitis and the development as well as progression of various respiratory diseases has been the subject of sustained scientific investigation over several decades. This review is an attempt to understand the existing association between periodontitis and TB.

### Epidemiology and clinical correlates

Observational and clinical data consistently indicate a notable co-occurrence of periodontitis and TB. MohanaSundaram et al. [14] emphasise that individuals with TB frequently exhibit periodontitis, proposing biological links involving shared inflammatory cytokines and immune system exhaustion. However, these associations arise from varied populations with potential confounders such as smoking and socioeconomic factors. Ojha et al. [16] reported that over two-thirds of patients with pulmonary diseases had periodontitis, though TB-specific associations were less clear due to mixed signals within lung disease cohorts. Sheereen et al. [17] highlight that oral TB lesions, which may mimic periodontal disease, require careful differential diagnosis in cases of persistent periodontal inflammation. Earlier clinical research by Sharma et al. [9] documented poorer periodontal health among TB patients compared to controls, underpinning much of the current evidence base despite limitations in sample size and study era.

### Microbiome & pathways

Emerging research has begun to elucidate how TB influences the oral microbiome and periodontal environment. Shahzad et al. [10] conducted the first profiling of oral microbiota in treatment-naïve TB cohorts, demonstrating significant dysbiosis compared to non-TB controls, suggesting the oral ecosystem is altered at TB onset. Zhang et al. [18] provide a broad synthesis of interactions between periodontitis and respiratory diseases, including TB, framing the role of systemic inflammation and oral pathogen aspiration in disease progression. Liu et al. [19] offer mechanistic insights on oral microbiome alterations affecting mucosal immunity and susceptibility to infections, which can inform TB–periodontitis interplay despite their focus not being TB-exclusive.

### Public health/practice and behavioural

From a public health perspective, the association between poor oral health and TB has important implications. A narrative review in the *LNH Journal* [15] advocates for integrating periodontal care into TB control programmes, underscoring the potential to reduce respiratory infection complications through oral hygiene. The *IJDMSR* [20] programme evaluation highlights that TB patients frequently present with more severe gingival and periodontal inflammation, and demonstrates the feasibility of oral health behaviour interventions during TB treatment. Nonetheless, methodological limitations in these implementation studies warrant further rigorous evaluation.

### Cross-cutting evidence

While systematic reviews and meta-analyses confirm a general link between periodontitis and respiratory diseases, they rarely isolate TB-specific effects. A recent meta-analysis (2022–2023) revealed consistent associations between periodontal disease and worsened pulmonary outcomes, supporting the hypothesis that chronic oral inflammation may influence respiratory conditions, including TB.

### Current interpretation and future directions

The current body of evidence suggests convergence rather than confirmed causality between TB and periodontitis. Clinical co-occurrence, oral TB lesions, and microbiome dysbiosis collectively point to complex bi-directional interactions where TB may exacerbate periodontal deterioration through immune modulation, while periodontal inflammation could contribute to systemic inflammatory burden impacting lung health. Clinicians should consider TB in cases of atypical or non-healing periodontal lesions and coordinate care accordingly. Longitudinal studies tracking periodontal status before, during, and after TB treatment are needed to clarify causality and therapeutic potential. Additionally, rigorously designed research addressing confounders such as smoking, diabetes, and socioeconomic factors is essential.

Microbiome and immune multi-omics approaches are a promising frontier for understanding pathogen–host interactions in the oral cavity during TB infection and treatment. Implementation research embedding periodontal screening and education within TB treatment programmes could improve clinical adherence, nutritional status, and patient quality of life, warranting further pilot studies.

## Conclusion

The existing literature reveals a compelling yet preliminary association between periodontitis and TB, characterised by heightened prevalence and severity of periodontal disease among TB patients, alongside shared pathways of chronic inflammation, microbial dysbiosis, and immune dysregulation. Observational studies, including case-control and cross-sectional designs, consistently demonstrate elevated probing pocket depths, bleeding on probing, and gingival indices in TB cohorts compared to controls, though heterogeneity in methodologies and confounders like smoking, HIV co-infection, and socioeconomic status preclude definitive causality. This scoping review underscores the bi-directional potential wherein periodontal inflammation may amplify systemic inflammatory burdens exacerbating TB progression, while *M. tuberculosis* dissemination could impair oral mucosal immunity and periodontal homeostasis. Future research should focus on long-term studies that follow patients before, during, and after TB treatment to better understand how TB and periodontitis affect each other. Studies using advanced techniques to examine the bacteria and immune responses in the mouth during TB infection could clarify how the two diseases interact.

### Practical implications for clinicians and public health programmes

Healthcare providers should incorporate routine screening for periodontitis in TB patients and consider early dental consultations to manage oral biofilms aggressively given the high co-occurrence and lesion overlap. Conversely, dental practitioners should maintain a high index of suspicion for TB in patients with persistent or unusual gingival ulcers, especially when accompanied by systemic symptoms or TB exposure history. Public health strategies may benefit from integrating oral health education within Directly Observed Treatment, Short-course (DOTS) and National Tuberculosis Control Programme (NTCP) workflows to promote better oral hygiene and potentially improve overall treatment outcomes.
